# O.4.3-1 Girls with low motor competence may be particularly at risk of insufficient skeletal loading; latent profiles analysis of a four-year follow-up of Finnish adolescents

**DOI:** 10.1093/eurpub/ckad133.187

**Published:** 2023-09-11

**Authors:** Timo Rantalainen, Arto Gråsten, Eero A Haapala, Francisco B Ortega, Mikko Huhtiniemi, Timo Jaakkola

**Affiliations:** Faculty of Sport and Health Sciences, University of Jyväskylä, Finland; Faculty of Sport and Health Sciences, University of Jyväskylä, Finland; United Arab Emirates University, College of Education, Al Ain, Abu Dhabi, United Arab Emirates; Faculty of Sport and Health Sciences, University of Jyväskylä, Finland; Faculty of Sport and Health Sciences, University of Jyväskylä, Finland; Department of Physical Education and Sports, Faculty of Sport Sciences, University of Granada, Granada, Spain; timo.rantalainen@jyu.fi; Faculty of Sport and Health Sciences, University of Jyväskylä, Finland; Faculty of Sport and Health Sciences, University of Jyväskylä, Finland

## Abstract

**Purpose:**

Adequate skeletal loads during the adolescent growth spurt are elemental in building a robust skeleton. Adolescents with poorer motor competence are less active and develop a less robust skeleton compared to their peers with better develop motor competence. However, few longitudinal examinations of motor competence and skeletal loading through adolescence have been reported. Therefore, the purpose of the present study was to explore the association between motor competence and free-living bone-generating (osteogenic) skeletal load trajectories among adolescents.

**Methods:**

A total of N = 562 5^th^ graders (11 to 13 years-of-age) were initially included in the sample and were followed up annually for four years from 2017 to 2020 (N = 239 in 2020). The children were asked to wear a waist-worn accelerometer for 7 days and motor competence was assessed with 5-leap and side-to-side jumping tests once a year. Latent profiles were identified based on motor competence, and skeletal loading was estimated using an osteogenic index, which combines the number of loading peaks in particular intensity categories akin to counting steps but with accounting for the magnitude of the impact.

**Results:**

Three motor competence groups arose from the data for both boys and girls and all groups exhibited persistent low, mid or high motor competence throughout the follow-up. The latent group with the low motor competence consistently exhibited the lowest skeletal loads (10 to 20% below the highest motor competence), and the highest motor competence group the highest with the mid-group in between. All latent groups declined in skeletal loads over the follow-up (from osteogenic index 420 SD[80] to 300[90]) with no difference in the steepness of the decline among boys. However, the lowest motor competence group among girls had a slightly steeper decline in skeletal loads than the other groups.

**Conclusions:**

the finding that motor competence is associated with skeletal loading among adolescents was confirmed in this longitudinal study. Notably, girls with the lowest motor competence had the steepest decline in skeletal loading over the follow-up. Policies aiming to improve fundamental movement skills could be expected to improve skeletal development particularly among less motor competent girls.

